# Association Between Oxidative Stress Biomarkers and Sperm DNA Damage in Idiopathic and Unexplained Male Infertility

**DOI:** 10.3390/biology15100802

**Published:** 2026-05-19

**Authors:** Kenza Berrada, Asmaa Serbouti, Abderrahmane Sadek, Yasmine Touhamia, Mohieddine Moumni, Noureddine Louanjli, Rachid Aboutaieb

**Affiliations:** 1 Laboratory of Sexual and Reproductive Health, Faculty of Medicine and Pharmacy, Hassan II University, Casablanca 20360, Morocco; 2 Biotechnology and Bioresources Valorization Laboratory, Biology Department, Faculty of Sciences, Moulay Ismail University, Meknes 11201, Morocco; 3 Health, Environment and Biotechnology Laboratory, Faculty of Sciences Ain Chock, Hassan II University, Casablanca 20100, Morocco; 4 Labomac Laboratory of Medical Analysis and Reproductive Biology, 40 Prince Moulay Abdallah Street, Casablanca 20000, Morocco; 5 Department of Urology, Ibn Rochd University Hospital Center, Casablanca 20100, Morocco

**Keywords:** oxidative stress, sperm DNA fragmentation, infertile men, sperm decondensation malondialdehyde, antioxidant enzyme

## Abstract

(1) Background: Oxidative stress (OS) has been extensively associated with male infertility, contributing to its pathophysiology in approximately 30–80% of affected individuals. Excessive reactive oxygen species (ROS) levels trigger a cascade of interconnected processes, including lipid peroxidation, DNA damage, and mitochondrial dysfunction, ultimately impairing sperm quality. (2) Methods: The present study was designed to examine the relationship between seminal oxidative stress biomarkers such as superoxide dismutase (SOD), catalase (CAT), and malondialdehyde (MDA) and sperm DNA integrity in men with unexplained (UMI) and idiopathic (IMI) male infertility. The study population comprised 235 participants, who were divided into three groups: fertile controls (*n* = 78), UMI (*n* = 88), and IMI (*n* = 69). Semen analysis was performed according to WHO criteria. Sperm DNA fragmentation index (DFI) was assessed using the TUNEL assay, while sperm decondensation index (SDI) was evaluated by aniline blue staining. Seminal OS biomarkers were measured in seminal plasma using spectrophotometric methods. (3) Results: DFI and SDI were significantly increased in infertile groups compared with fertile controls (*p* < 0.001), exceeding the clinical thresholds of 30% and 15%, respectively. Antioxidant enzyme activities (SOD and CAT) were significantly reduced, whereas MDA levels were significantly elevated, particularly in the IMI group (*p* < 0.001). Correlation analysis revealed strong positive associations between MDA and both DFI (r = 0.76, *p* < 0.001) and SDI (r = 0.80, *p* < 0.001). Additionally, MDA, DFI, and SDI were negatively correlated with semen parameters, whereas SOD and CAT were positively correlated with sperm quality (*p* < 0.001). (4) Conclusions: Our findings emphasize the critical role of oxidative stress and sperm DNA integrity as potential biomarkers for the diagnosis and evaluation of UMI and IMI.

## Simple Summary

Oxidative imbalance is widely recognized as a major contributor to male infertility. In this study, we evaluated the relationship between oxidative stress biomarkers and sperm DNA integrity in 235 men, including a group of fertile men and patients with unexplained and idiopathic male infertility. Our results demonstrate that infertile men are characterized by elevated levels of DNA damage and oxidative stress levels, along with reduced antioxidant defenses. Strong associations were observed between lipid peroxidation and sperm DNA damage. Our findings highlight the potential clinical value of oxidative stress and sperm DNA integrity markers in the diagnostic evaluation of idiopathic and unexplained male infertility.

## 1. Introduction

Infertility remains a major global health challenge, affecting approximately 15% of couples, with male-related causes accounting for 40–50% of cases, including nearly 30% classified as idiopathic (IMI) [1,2]. IMI is defined by abnormal semen parameters in the absence of an identifiable cause [3]. In contrast, unexplained male infertility (UMI) accounts for approximately 15–40% of infertile men, who present normal semen parameters despite the absence of identifiable clinical abnormalities (such as infections, varicocele, or hormonal abnormalities) and the exclusion of female infertility factors, yet fail to achieve conception [3,4]. This further suggests that conventional semen analysis may not fully reflect sperm functional competence [5].

Although the mechanisms responsible for idiopathic and unexplained male infertility are not yet fully elucidated, oxidative stress (OS) is now widely acknowledged as a key contributor. It is implicated in approximately 30–80% of cases [2,6] and reflects an imbalance between reactive oxygen species (ROS) generation and antioxidant defenses [7]. Through multiple interconnected mechanisms, excessive ROS promote lipid peroxidation, DNA damage, and mitochondrial dysfunction, ultimately impairing sperm quality [8].

Spermatozoa are particularly vulnerable to oxidative damage due to their unique structural and functional characteristics. Their plasma membrane is enriched in polyunsaturated fatty acids, which enhance membrane fluidity but also increase susceptibility to lipid peroxidation [8,9]. Consequently, oxidative reactions generate reactive aldehydes such as malondialdehyde (MDA) and 4-hydroxynonenal (4-HNE), which can compromise membrane integrity and impair cellular functions [10]. Although sperm DNA is highly compacted by protamines, it has limited DNA repair capacity, making it highly sensitive to oxidative damage [11]. Under oxidative stress, reactive oxygen species induce DNA damage, leading to increased DNA fragmentation and abnormal chromatin structure, thereby reducing fertilization potential [12]. Additionally, oxidative stress impairs mitochondrial function by reducing ATP production, negatively affecting sperm motility and viability [13,14].

Moreover, during maturation, spermatozoa undergo profound morphological changes, including cytoplasmic loss. As a result, their intracellular antioxidant capacity is limited, and they rely largely on antioxidants present in seminal plasma to counteract oxidative stress [15].

Despite normal semen parameters, unexplained male infertility persists, suggesting hidden alterations not detected by conventional semen analysis, potentially involving oxidative stress and sperm DNA damage. Similar underlying mechanisms may also contribute to idiopathic male infertility. However, the specific characterization of these alterations, particularly in normozoospermic infertile men, remains insufficiently explored. Therefore, this study aimed to provide new insights by investigating the association between seminal oxidative stress biomarkers, including superoxide dismutase (SOD), catalase (CAT), and malondialdehyde (MDA), sperm DNA integrity, and semen parameters in men with idiopathic and unexplained infertility.

## 2. Materials and Methods

### 2.1. Study Design

**Figure 1:**
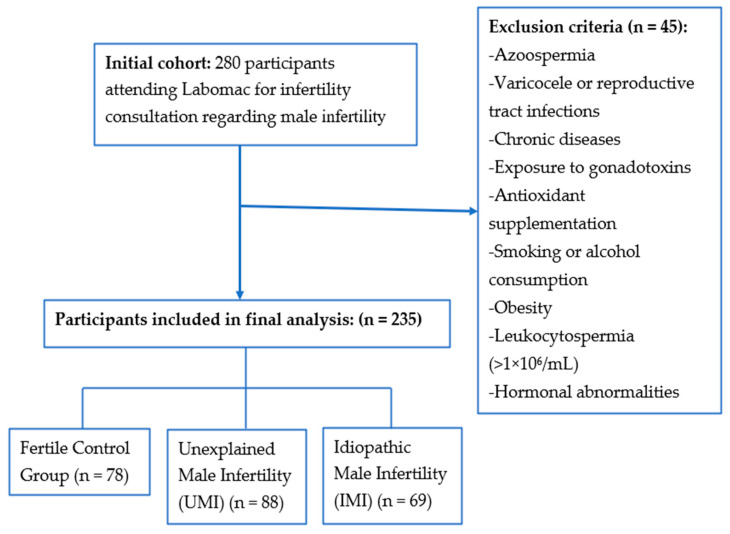
Flowchart illustrating participant selection, exclusion criteria, and group classification.

A total of 280 volunteers aged 25–45 years were initially enrolled in this prospective controlled study conducted at the Laboratory of Medical Analysis and Reproductive Biology (Labomac) in Casablanca, Morocco. The study was carried out from April 2022 to December 2025 in collaboration with the Health, Environment and Biotechnology Laboratory, Faculty of Sciences, Hassan II University of Casablanca, Morocco. After applying the inclusion and exclusion criteria, 235 participants were retained and classified into three groups: a fertile control group (78), an unexplained male infertility group (UMI) (88), and an idiopathic male infertility group (IMI) (69) (Figure 1). The control group consisted of fertile men with recently proven fertility who had fathered a child within the year prior to enrollment and served as the reference group. In addition, female related factors were excluded in all couples to ensure that infertility was attributable to the male partner.

### 2.2. Ethics

The study protocol was approved by the Ethics Committee of the Ibn Rochd University Hospital Center, Casablanca, Morocco (Ref: 2022/60.CEH.S.KS1.59; File No. 12/2022). All procedures were carried out in accordance with the ethical principles of institutional and national research committees, as well as the Declaration of Helsinki (1975, revised in 2013). Written informed consent was obtained from all participants before their inclusion in the study.

### 2.3. Semen Analysis

Semen analysis was conducted in accordance with the World Health Organization (WHO) guidelines (2021) [16]. Semen samples were collected following 3–5 days of abstinence and were allowed to liquefy at 37 °C for approximately 60 min. Seminal parameters, including volume, concentration, motility, vitality, and morphology, were assessed using a computer-assisted semen analyzer (CASA; Microptic, Barcelona, Spain) [17]. Sperm motility was classified as progressive (PR), non-progressive (NP), or immotile (IM). Vitality was evaluated using eosin staining, and morphology was assessed after Shorr-Hematoxylin staining according to standard criteria. All semen analyses were performed in duplicate for each participant to ensure accuracy and reproducibility.

### 2.4. DNA Fragmentation Index (DFI)

Sperm DNA fragmentation was assessed using the TUNEL assay (Terminal deoxynucleotidyl transferase dUTP nick end labeling) with the In Situ Cell Death Detection Kit, Fluorescein (Roche Diagnostics GmbH, Mannheim, Germany; REF 11684795910), following the manufacturer’s instructions. This method relies on the enzymatic activity of terminal deoxynucleotidyl transferase (TdT), which facilitates the incorporation of fluorescein labeled dUTP at the 3′-OH termini of DNA strand breaks [18].

Semen samples were washed twice with phosphate-buffered saline (PBS; Sigma-Aldrich, Gillingham, UK) and adjusted to a final concentration of 2 × 10^7^ cells/mL. The cell suspension was then fixed in 2% formaldehyde in PBS for 30 min at room temperature. Following fixation, samples were rinsed twice with PBS and centrifuged at 200× *g* for 10 min. Aliquots of the cell suspension were then smeared onto glass slides and air-dried.

The samples were then incubated with the TUNEL reaction mixture (TdT and fluorescein labeled dUTP) at 37 °C for 45 min according to the manufacturer’s instructions. Prepared slides were examined under a fluorescence microscope. For each slide, at least 500 sperm cells were counted, and DFI was calculated as the percentage of fluorescent sperm cells. Fragmented DNA fluoresced, whereas intact DNA showed no signal. DFI values exceeding 15% were interpreted as abnormal and associated with impaired sperm nuclear integrity [19].

### 2.5. Sperm Decondensation Index (SDI)

Assessment of chromatin condensation was performed using aniline blue staining based on the method reported by Kim et al. [20]. Briefly, sperm smears were prepared from the cell suspension and air dried. Fixation and permeabilization of spermatozoa were performed using the same protocol as described for sperm DNA fragmentation. Subsequently, smears were stained with 5% aniline blue solution (pH 3.5, 1 mL per slide) for 15 min at room temperature. After staining, slides were rinsed with tap water and air dried. The stained smears were then examined under a light microscope using ×100 magnification with oil immersion.

At least 500 spermatozoa were examined per sample. Sperm cells with dark blue staining, indicative of excessive histone retention and incomplete chromatin maturation, were identified as abnormal. The sperm DNA decondensation index (SDI) was calculated as the percentage of stained spermatozoa, with values ≥ 15% considered pathological.

After evaluating sperm DNA integrity, semen samples were centrifuged at 1000× *g* for 10 min. The supernatant (seminal plasma) was then collected and stored at −20 °C for subsequent analysis of oxidative stress biomarkers.

### 2.6. Malondialdehyde (MDA) Assay

Lipid peroxidation levels were assessed by measuring substances reacting with thiobarbituric acid (TBARS) according to the method described by Samokyszyn and Marnett (1990) [21]. An aliquot of 100 µL of seminal plasma was combined with a reagent solution containing 0.67% thiobarbituric acid (TBA) and 10% trichloroacetic acid prepared in 0.25 M hydrochloric acid. The mixture was incubated in a boiling water bath at 100 °C for 15 min, then rapidly cooled on ice and centrifuged at 1000× *g* for 10 min. The absorbance of the supernatant was recorded at 535 nm. MDA levels were calculated based on an extinction coefficient of 1.56 × 10^5^ M^−1^·cm^−1^ and reported in nmol/mL.

### 2.7. Catalase (CAT) Activity

The activity of catalase (CAT) was assessed using a spectrophotometric method based on Aebi (1984) [22]. Briefly, the assay mixture consisted of 50 mM phosphate buffer (pH 7.0) combined with 50 µL of seminal plasma. The reaction was initiated by the addition of 7.5 mM H_2_O_2_, and catalase activity was determined by monitoring the decomposition of hydrogen peroxide at 240 nm. Specific activity was calculated using the molar extinction coefficient of H_2_O_2_ (ε = 40 M^−1^·cm^−1^) and expressed as Units per mg protein.

### 2.8. Superoxide Dismutase (SOD) Activity

Superoxide dismutase (SOD) activity was assessed following the method of Paoletti and Mocali (1990) [23]. The reaction mixture contained 5 mM EDTA, 2.5 mM MnCl_2_, 0.27 mM NADH, 3.9 mM 2-mercaptoethanol, and 50 mM phosphate buffer (pH 7.0), along with 50 µL of seminal plasma. Enzyme activity was monitored spectrophotometrically at 340 nm by following the oxidation of NADH. Activity was calculated using the molar extinction coefficient of NADH (ε = 6220 M^−1^·cm^−1^) and expressed as Units per milligram of protein.

### 2.9. Protein Assay

Protein levels were quantified using the Bradford assay (1976), with bovine serum albumin (BSA) serving as the standard [24]. This colorimetric assay is sensitive and suitable for detecting low protein concentrations in the microgram range.

### 2.10. Statistical Analysis

Statistical analyses were performed using RStudio software (version 4.4.2). Data were summarized and visualized using boxplots generated with the ggplot2 package. The normality of each variable was evaluated using the Shapiro–Wilk test, and the homogeneity of variances was assessed using Levene’s test. For comparisons between groups, one-way analysis of variance (ANOVA), followed by Tukey’s honestly significant difference (HSD) post hoc test, was performed when data were normally distributed and variances were homogeneous (*p* ≥ 0.05). When the assumptions of normality or homogeneity of variances were not met (*p* < 0.05), the Kruskal–Wallis test was used, followed by Dunn’s post hoc test with Benjamini–Hochberg adjustment.

Correlations were analyzed to investigate the associations among oxidative stress biomarkers, semen parameters, and sperm DNA integrity.

Spearman’s rank correlation was applied, with statistical significance defined at *p* < 0.05.

## 3. Results

### 3.1. Data Summary of Participants According to Semen Parameters, Sperm DNA Integrity, and Oxidative Stress Biomarkers

**Figure 2:**
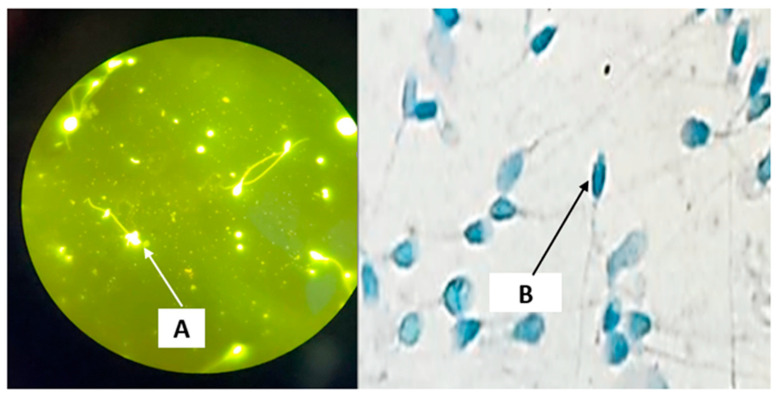
Representative microscopic images illustrating sperm DNA fragmentation and chromatin decondensation. (**A**) TUNEL assay indicating spermatozoa with fragmented DNA (green fluorescence). (**B**) Aniline blue staining showing spermatozoa with decondensed chromatin (dark blue staining).

**Figure 3:**
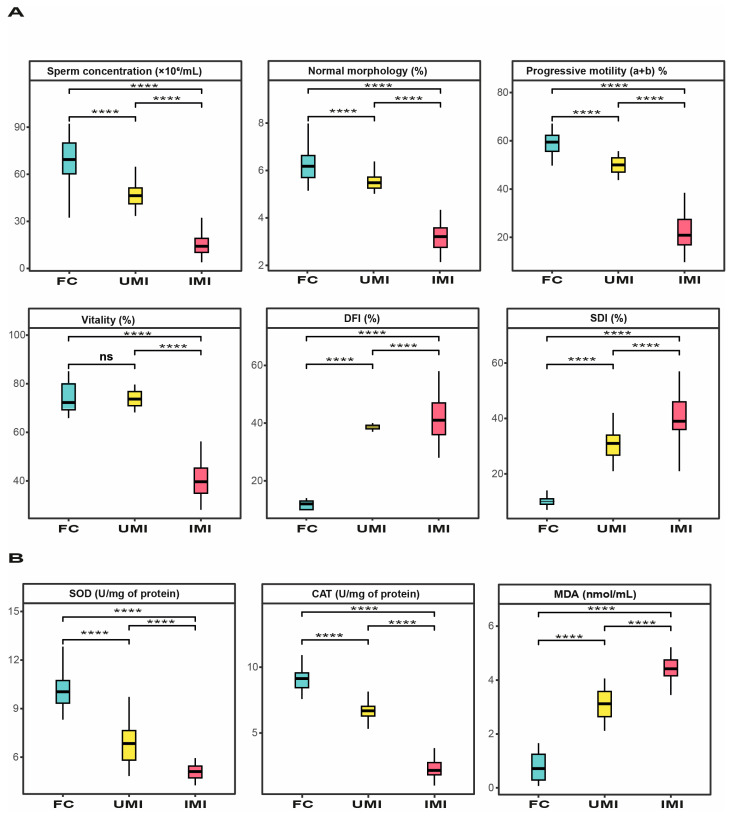
Comparative distribution of semen parameters, sperm DNA integrity indices (DFI and SDI), and oxidative stress biomarkers among fertile controls (FC), unexplained male infertility (UMI), and idiopathic male infertility (IMI) groups. Data are presented as boxplots indicating the median, interquartile range (box), and minimum–maximum values (whiskers). Statistical comparisons were performed using the Kruskal–Wallis test followed by Dunn’s post hoc test with Benjamini–Hochberg correction. Significance levels are indicated as follows: ns, not significant; **** *p* < 0.0001.

Representative microscopic images illustrating sperm DNA integrity are presented in Figure 2, whereas descriptive statistics for age, semen parameters, sperm DNA integrity, and oxidative stress biomarkers in control and infertile groups are shown in Table 1 and Figure 3. This study included 235 volunteers. The age of participants ranged from 33.05 ± 5.07 to 33.62 ± 4.62 years, with no significant difference between fertile and infertile groups (*p* > 0.05). The abstinence period was comparable across groups (3–5 days). Ejaculate volume remained comparable (approximately 3.0 mL), with no significant differences detected (*p* > 0.05). Leukocyte concentrations were below 1 × 10^6^/mL in all groups and did not differ significantly (*p* > 0.05), indicating the absence of leukocytospermia and suggesting no inflammatory response.

In contrast, sperm concentration, normal morphology, progressive motility, and vitality were significantly lower in infertile men than in fertile controls (*p* < 0.05). Although semen parameters in the unexplained male infertility (UMI) group remained within WHO (2021) reference ranges, they were consistently lower than those of fertile controls. These alterations were more pronounced in the idiopathic male infertility (IMI) group, which exhibited the most marked impairment in sperm quality.

In terms of sperm DNA integrity and oxidative stress biomarkers, infertile groups showed significantly elevated levels of DNA fragmentation index (DFI) and sperm decondensation index (SDI) compared to fertile controls (*p* < 0.001). Notably, DFI values exceeded the clinical threshold of 30% in infertile patients, indicating impaired sperm DNA integrity. These findings were corroborated by microscopic observations (Figure 2), where TUNEL assay revealed spermatozoa with fragmented DNA (green fluorescence), while aniline blue staining showed increased chromatin decondensation (dark blue staining).

A significant reduction in antioxidant enzyme activities, including superoxide dismutase (SOD) and catalase (CAT), was observed in infertile men compared to controls (*p* < 0.001), with the lowest levels observed in the IMI group. In contrast, lipid peroxidation, assessed by malondialdehyde (MDA) levels, was significantly increased in infertile groups (*p* < 0.05), reaching its highest levels in IMI patients, followed by UMI patients. Overall, the IMI group exhibited the most pronounced alterations, followed by the UMI group, indicating a progressive deterioration in sperm quality.

Kruskal–Wallis analysis confirmed highly significant differences among groups for all biomarkers, with large effect sizes (ε^2^ = 0.69–0.87). Post hoc analysis using Dunn’s test with Benjamini–Hochberg correction demonstrated significant differences between all groups (*p* < 0.05), further supporting a gradient from fertile controls to UMI and IMI patients.

### 3.2. Correlations Between Semen Parameters, Sperm DNA Integrity Indices (DFI and SDI), and Oxidative Stress Biomarkers

**Figure 4:**
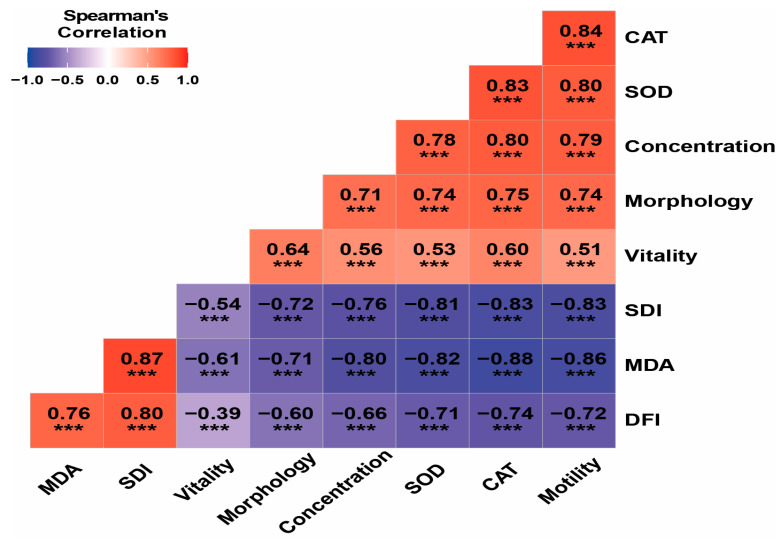
Spearman correlation matrix illustrating the relationships between semen parameters, sperm DNA integrity indices (DFI and SDI), and oxidative stress biomarkers. Statistical significance was defined as follows: *** *p* < 0.001. The color gradient represents the strength and direction of correlations, with red indicating positive correlations and blue indicating negative correlations, and color intensity reflecting the magnitude of the correlation coefficient.

Spearman correlation analysis revealed strong and significant associations between oxidative stress biomarkers, sperm DNA integrity indices, and semen parameters (Figure 4). Positive correlations were identified between antioxidant enzymes (SOD and CAT) and semen quality parameters, including sperm concentration, motility, morphology, and vitality (r = 0.74–0.84, *p* < 0.001). In contrast, DNA fragmentation index (DFI), sperm decondensation index (SDI), and malondialdehyde (MDA) levels were negatively correlated with semen parameters (r ranging from −0.39 to −0.88, *p* < 0.001), indicating that increased oxidative stress and DNA damage are associated with impaired sperm quality. Notably, strong positive associations were observed between MDA and both DFI and SDI (r = 0.76 and r = 0.80, respectively; *p* < 0.001), suggesting a close relationship between lipid peroxidation and sperm DNA damage. In contrast, SOD and CAT activities were strongly negatively correlated with DFI, SDI, and MDA levels (r ranging from −0.71 to −0.88, *p* < 0.001), highlighting their protective role against oxidative stress-induced sperm damage.

## 4. Discussion

Oxidative stress is a major contributor to impaired sperm function and male reproductive dysfunction, mainly driven by the excessive generation of reactive oxygen species (ROS) [25]. These reactive molecules initiate lipid peroxidation and induce both nuclear and mitochondrial DNA damage, alterations that are not detectable by conventional semen analysis [26]. In this context, the present study aimed to investigate the relationship between oxidative stress biomarkers, sperm DNA integrity, and semen parameters in men with idiopathic (IMI) and unexplained infertility (UMI), compared with fertile control men (FC).

Across all groups, no statistically significant differences were observed in age, abstinence duration, semen volume, or leukocyte counts (*p* > 0.05; Table 1), indicating that these variables do not explain the observed alterations. In contrast, semen parameters (sperm concentration, normal morphology, progressive motility, and vitality) were significantly impaired in infertile groups, particularly in the IMI group (*p* < 0.001). Although values in the UMI group remained within the normal reference ranges defined by the WHO (2021), they were consistently lower than those observed in fertile men.

These results are in agreement with previous reports indicating altered semen parameters in idiopathic infertility, while remaining within normal reference ranges in unexplained infertility [27,28]. This pattern highlights the limitations of conventional semen analysis, as patients with unexplained infertility may present normal results despite an inability to conceive, suggesting that routine evaluation alone cannot detect underlying functional abnormalities and supporting the role of non-inflammatory factors such as oxidative stress [29].

Importantly, our findings provide novel insight into unexplained male infertility by demonstrating that normozoospermic infertile men (UMI) may exhibit significant oxidative stress and sperm DNA damage despite apparently normal semen profiles, highlighting the need for additional diagnostic biomarkers.

As previously reported, all infertile groups exhibited a marked increase in sperm DNA fragmentation index (DFI > 30%) and sperm DNA decondensation index (SDI > 15%) compared to the control group (DFI < 15% and SDI < 15%) (Table 1, Figure 3). Our findings are consistent with previous studies reporting elevated sperm DNA fragmentation in men with UMI, suggesting that DNA damage may contribute to its underlying mechanisms [30,31,32]. Similarly, increased DNA fragmentation has also been reported in men with IMI, as well as in asthenozoospermic and oligozoospermic patients, further supporting its role in male infertility [33,34]. We also observed significant correlations, with DFI, SDI, and MDA negatively associated with semen parameters, while strong positive correlations were found between MDA and both DFI and SDI, highlighting the detrimental effect of oxidative stress on sperm quality and DNA integrity (*p* < 0.001). Our results are in line with earlier studies, including recent findings showing a positive association between sperm DNA fragmentation index (DFI) and MDA levels (*p* < 0.01), as well as a negative correlation with total antioxidant capacity (*p* < 0.01). In addition, DFI was negatively associated with sperm survival, concentration, and progressive motility (PR%) (*p* < 0.01), further supporting the link between oxidative stress and impaired sperm function [35].

In this context, reactive oxygen species (ROS) can lead to DNA strand breaks, impair chromatin packaging, and promote epigenetic dysregulation, ultimately contributing to reduced sperm quality and male infertility [36]. Due to their weak antioxidant defenses and limited DNA repair capacity, spermatozoa are highly prone to oxidative damage [11]. Under these conditions, ROS metabolites attack DNA bases, particularly guanine, as well as the phosphodiester backbone, leading to structural instability and DNA fragmentation [37,38]. Sperm DNA integrity is essential for the accurate transmission of genetic information and the development of healthy offspring, and the male gamete also plays a crucial role in proper embryo development [39]. However, a study has reported higher sperm DNA fragmentation in normozoospermic men compared with those presenting semen abnormalities [40]. These variations may be related to differences in study populations, methodologies, and the use of fertile controls in our study.

Regarding oxidative stress biomarkers, MDA levels were significantly elevated in all infertile groups, with the highest values observed in the IMI group, reflecting enhanced lipid peroxidation. These findings in accordance with previous studies reporting increased MDA levels in patients with impaired semen quality, including asthenozoospermic and teratozoospermic individuals [41,42], as well as in men with UMI despite normal semen parameters [43]. Moreover, MDA levels were negatively associated with semen parameters as well as with antioxidant enzymes (SOD and CAT). This effect can be explained by the susceptibility of spermatozoa to lipid peroxidation. As this process progresses, a substantial loss of membrane fatty acids occurs, leading to decreased membrane fluidity, increased nonspecific ion permeability, and inactivation of membrane-bound receptors and enzymes [44,45]. This vulnerability is largely due to the high content of polyunsaturated fatty acids in the sperm membrane, making it particularly sensitive to oxidative damage. It has also been shown that exposure of human spermatozoa to extracellularly generated ROS induces a loss of motility, which is directly correlated with the level of lipid peroxidation in spermatozoa [46]. Furthermore, these alterations impair other membrane dependent functions, including sperm–oocyte fusion and the acrosome reaction, ultimately compromising fertilization capacity [47].

Concerning antioxidant enzymes (SOD and CAT), we observed a significant decrease in their activity across all infertile groups compared with controls, indicating a weakened defense against ROS. These results are in agreement with previous studies reporting reduced antioxidant levels in infertile men [28,48]. This coordinated decrease may reflect the synergistic action of these enzymes, whereby SOD converts superoxide radicals into hydrogen peroxide, which is subsequently detoxified by CAT into oxygen and water [49].

This study has some limitations. Only selected oxidative stress biomarkers and sperm DNA integrity parameters were assessed, while other relevant markers were not evaluated. In addition, participants were recruited from a single center.

## 5. Conclusions

In summary, our findings indicate that oxidative stress contributes significantly to male infertility by adversely affecting sperm quality and DNA integrity. Infertile men, particularly those with idiopathic infertility, exhibited increased levels of lipid peroxidation (MDA), increased sperm DNA fragmentation (DFI) and chromatin decondensation (SDI), along with reduced antioxidant defenses (SOD and CAT). Furthermore, significant correlations were observed between oxidative stress biomarkers and sperm DNA damage, with MDA positively correlated with DFI and SDI, while exhibiting negative correlations with semen parameters such as sperm concentration, normal morphology, progressive motility and vitality. In contrast, antioxidant enzymes (SOD and CAT) were positively associated with sperm quality parameters, highlighting the critical balance between oxidative stress and antioxidant defense in maintaining sperm function.

Notably, men with unexplained infertility presented normal semen parameters despite underlying oxidative stress and DNA damage, highlighting the limitations of conventional semen analysis. Importantly, these findings provide novel insight into unexplained male infertility by demonstrating that normal semen parameters do not necessarily reflect normal sperm function. These findings support the potential value of incorporating oxidative stress biomarkers and sperm DNA integrity assessments into the routine evaluation of male infertility.

## Figures and Tables

**Table 1:** Semen parameters, sperm DNA integrity indices (DFI and SDI), and oxidative stress biomarkers among fertile controls and infertile men.

Parameters	Fertile Control *N* = 78	UMI *N* = 88	IMI *N* = 69	*p*-Value
**Age (years)**	33.05 ± 5.07 ^a^	33.27 ± 4.47 ^a^	33.62 ± 4.62 ^a^	**0.8**
**Abstinence (days)**				**>0.9**
**3**	16 (21%)	17 (19%)	14 (20%)	
**4**	47 (60%)	53 (60%)	41 (59%)	
**5**	15 (19%)	18 (20%)	14 (20%)	
**Semen volume (mL)**	3.08 ± 0.43 ^a^	3.04 ± 0.38 ^a^	3.04 ± 0.38 ^a^	**0.8**
**Sperm concentration (×10^6^/mL)**	68.42 ± 14.86 ^a^	47.65 ± 9.71 ^b^	15.26 ± 7.10 ^c^	**<0.001**
**Normal morphology (%)**	6.19 ± 0.65 ^a^	5.55 ± 0.43 ^b^	3.19 ± 0.54 ^c^	**<0.001**
**Progressive motility (a + b) %**	59.18 ± 4.29 ^a^	50 ± 3.49 ^b^	22.26 ± 8.19 ^c^	**<0.001**
**Vitality (%)**	74.15 ± 5.93 ^a^	74 ± 3.38 ^a^	41 ± 8.19 ^b^	**<0.001**
**Leukocytes (×10^6^/mL)**	0.47 ± 0.23 ^a^	0.49 ± 0.21 ^a^	0.48 ± 0.21 ^a^	**0.8**
**DFI %**	11.50 ± 1.30 ^a^	38.27 ± 1.81 ^b^	42.21 ± 6.59 ^c^	**<0.001**
**SDI %**	10.03 ± 1.65 ^a^	30.07 ± 4.57 ^b^	40.52 ± 7 ^c^	**<0.001**
**SOD (U/mg of protein)**	10.12 ± 1.07 ^a^	6.71 ± 1.07 ^b^	5.09 ± 0.46 ^c^	**<0.001**
**CAT (U/mg of protein)**	9.07 ± 0.79 ^a^	6.67 ± 0.95 ^b^	2.28 ± 0.76 ^c^	**<0.001**
**MDA (nmol/mL)**	0.78 ± 0.50 ^a^	3.11 ± 0.56 ^b^	4.46 ± 0.40 ^c^	**<0.001**

Values are expressed as mean ± standard deviation. Bold values indicate statistically significant differences (*p* < 0.05). Different superscript letters (a–c) indicate statistically significant differences between groups (*p* < 0.05), whereas groups sharing the same letter do not differ significantly. Statistical comparisons were performed using the Kruskal–Wallis test followed by Dunn’s post hoc test with Benjamini–Hochberg adjustment. DFI: DNA fragmentation index; SDI: sperm decondensation index; SOD: superoxide dismutase; CAT: catalase; MDA: malondialdehyde.

## Data Availability

Data Availability Statement: All data relevant to this study are provided within the article.

## Abbreviations

The following abbreviations are used in this manuscript:

IMI	Idiopathic Male Infertility
UMI	Unexplained Male Infertility
OS	Oxidative Stress
ROS	Reactive Oxygen Species
MDA	Malondialdehyde
4-HNE	4-Hydroxynonenal
ATP	Adenosine Triphosphate
SOD	Superoxide Dismutase
CAT	Catalase
DFI	DNA Fragmentation Index
SDI	Sperm DNA Decondensation Index
TdT	Terminal deoxynucleotidyl transferase
dUTP	Deoxyuridine triphosphate
TBARS	Thiobarbituric Acid Reactive Substances
EDTA	Ethylenediaminetetraacetic acid
NADH	Nicotinamide adenine dinucleotide (reduced form)
